# Racial and Ethnic Differences in Older Adults’ Financial Capacity and Differential Effects of Clinical Factors

**DOI:** 10.1002/alz.094796

**Published:** 2025-01-09

**Authors:** Sarah Therrien, Oceanna Yueran Li, Feng Vankee Lin

**Affiliations:** ^1^ Stanford University, Stanford, CA USA

## Abstract

**Background:**

Financial capacity (FC) encompasses one’s ability to understand and complete financial tasks and is essential to older adults’ functional independence. FC is impacted by neuropsychological, neuropsychiatric, and everyday functioning factors in aging. In FC literature, much research does not include demographically representative samples. With a rapidly expanding proportion of older adults from racial and ethnic groups underrepresented in research, it is important to understand factors influencing independence in aging across all segments of the population. Some research shows several FC‐related clinical factors differ between non‐Hispanic white (NHW) and Black/African‐American (BAA) and Hispanic/Latino (HL) groups. Potential differential effects of these factors on groups’ FC are little understood. Here, we aim to understand racial and ethnic differences in FC and how clinical factors affect these relationships.

**Method:**

We used data from ADNI participants with normal cognition, MCI, or AD dementia who completed Financial Capacity Instrument and identified as NHW, BAA, or HL (total n = 900). The Neuropsychiatric Inventory (NPI), Everyday Cognition (ECog) Scale, and Memory and Executive Function (EF) composite scores were used to evaluate clinical factors. Analysis on group differences, main effect of FC, and moderation and mediation analyses were conducted with the clinical stage controlled.

**Result:**

BAA (Wald’s ^2^ = 15.06, p < .001), but not HL, had worse FC than NHW. In the entire sample there were significant correlations between FC and clinical factors (all ^2^ >7.60 and p<0.006). NPI was a significant moderator with FC in BAA was less impacted by NPI than in NHW (B = ‐0.49, SE = 0.19, Wald’s ^2^ = 6.84, p = .009). EF (B = ‐0.27, SE = 0.06, 95%CI:‐0.39, ‐0.15) and partner‐report ECog (B = ‐0.05, SE = 0.02, 95%CI: ‐0.09,‐0.01) were significant mediators for FC between BAA and NHW.

**Conclusion:**

There are key differences in FC between racial and ethnic groups, especially between BAA and NHW older adults, with neuropsychiatric and neuropsychological factors affecting FC differently between groups.

